# Spatiotemporal clusters of malaria cases at village level, northwest Ethiopia

**DOI:** 10.1186/1475-2875-13-223

**Published:** 2014-06-06

**Authors:** Kassahun Alemu, Alemayehu Worku, Yemane Berhane, Abera Kumie

**Affiliations:** 1 Department of Environmental and Occupational Health and Safety, Institute of Public Health, College of Medicine and Health Sciences, University of Gondar, Gondar, Ethiopia; 2 Department of Epidemiology and Biostatistics, School of Public Health, College of Health Sciences, Addis Ababa University, Addis Ababa, Ethiopia; 3 Addis Continental Institute of Public Health, Addis Ababa, Ethiopia; 4 Department of Environmental Health, School of Public Health, College of Health Sciences, Addis Ababa University, Addis Ababa, Ethiopia

**Keywords:** Altitude, Malaria, Spatial, Temporal, Village, Ethiopia

## Abstract

**Background:**

Malaria attacks are not evenly distributed in space and time. In highland areas with low endemicity, malaria transmission is highly variable and malaria acquisition risk for individuals is unevenly distributed even within a neighbourhood. Characterizing the spatiotemporal distribution of malaria cases in high-altitude villages is necessary to prioritize the risk areas and facilitate interventions.

**Methods:**

Spatial scan statistics using the Bernoulli method were employed to identify spatial and temporal clusters of malaria in high-altitude villages. Daily malaria data were collected, using a passive surveillance system, from patients visiting local health facilities. Georeference data were collected at villages using hand-held global positioning system devices and linked to patient data. Bernoulli model using Bayesian approaches and Marcov Chain Monte Carlo (MCMC) methods were used to identify the effects of factors on spatial clusters of malaria cases. The deviance information criterion (DIC) was used to assess the goodness-of-fit of the different models. The smaller the DIC, the better the model fit.

**Results:**

Malaria cases were clustered in both space and time in high-altitude villages. Spatial scan statistics identified a total of 56 spatial clusters of malaria in high-altitude villages. Of these, 39 were the most likely clusters (LLR = 15.62, p < 0.00001) and 17 were secondary clusters (LLR = 7.05, p < 0.03). The significant most likely temporal malaria clusters were detected between August and December (LLR = 17.87, p < 0.001). Travel away home, males and age above 15 years had statistically significant effect on malaria clusters at high-altitude villages.

**Conclusion:**

The study identified spatial clusters of malaria cases occurring at high elevation villages within the district. A patient who travelled away from home to a malaria-endemic area might be the most probable source of malaria infection in a high-altitude village. Malaria interventions in high altitude villages should address factors associated with malaria clustering.

## Background

Malaria transmission varies in space and time [1,2]. It is a multifactorial disease and the aetiological agent has a complex life cycle requiring an insect vector, and the attribution of trends to specific factors remains difficult because of multiple drivers, including micro-ecology, utilization of interventions, demographic and population movement [3], drug resistance, land-use change, and access to health facilities [4,5].

In highland areas with low endemicity, malaria transmission is highly variable and malaria acquisition risk for individuals is unevenly distributed even within a neighbourhood [6-8]. Many studies have been conducted to understand the spatiotemporal variation of malaria at areas below 2,000 m above sea level [9-14]. However, information about the spatiotemporal variation of malaria transmission risks in villages at altitude above 2,000 m is limited and poorly understood. As malaria transmission declines, understanding the heterogeneity in malaria transmission becomes crucial and creates opportunities for targeting interventions, while better methods are required in the field and in modelling. Understanding heterogeneity should focus [8] on individuals who contribute disproportionately to malaria transmission and other driving factors. Such targeted interventions are supremely important tools in malaria elimination efforts [15].

Recent studies employing a combination of geographical information systems (GIS) [13,16], scan statistics [17-19] and Bayesian modelling [20-22] have proven helpful in characterizing the spatial patterns of malaria and in determining linkages between spatial patterns and driving factors for malaria transmission risk. Improved understanding of spatiotemporal clustering of malaria in villages would be indispensable for government and malaria control and prevention partners to provide appropriate malaria interventions and resource allocation.

The aims of this study were to describe the spatial and temporal patterns of malaria transmission and identify drivers of the spatiotemporal patterns of malaria risk in high-altitude villages of northwest Ethiopia in an area of very low transmission intensity. The findings presented here have the potential to enhance the effectiveness of the national malaria control programme.

## Methods

### Study area and settings

**Figure 1:**
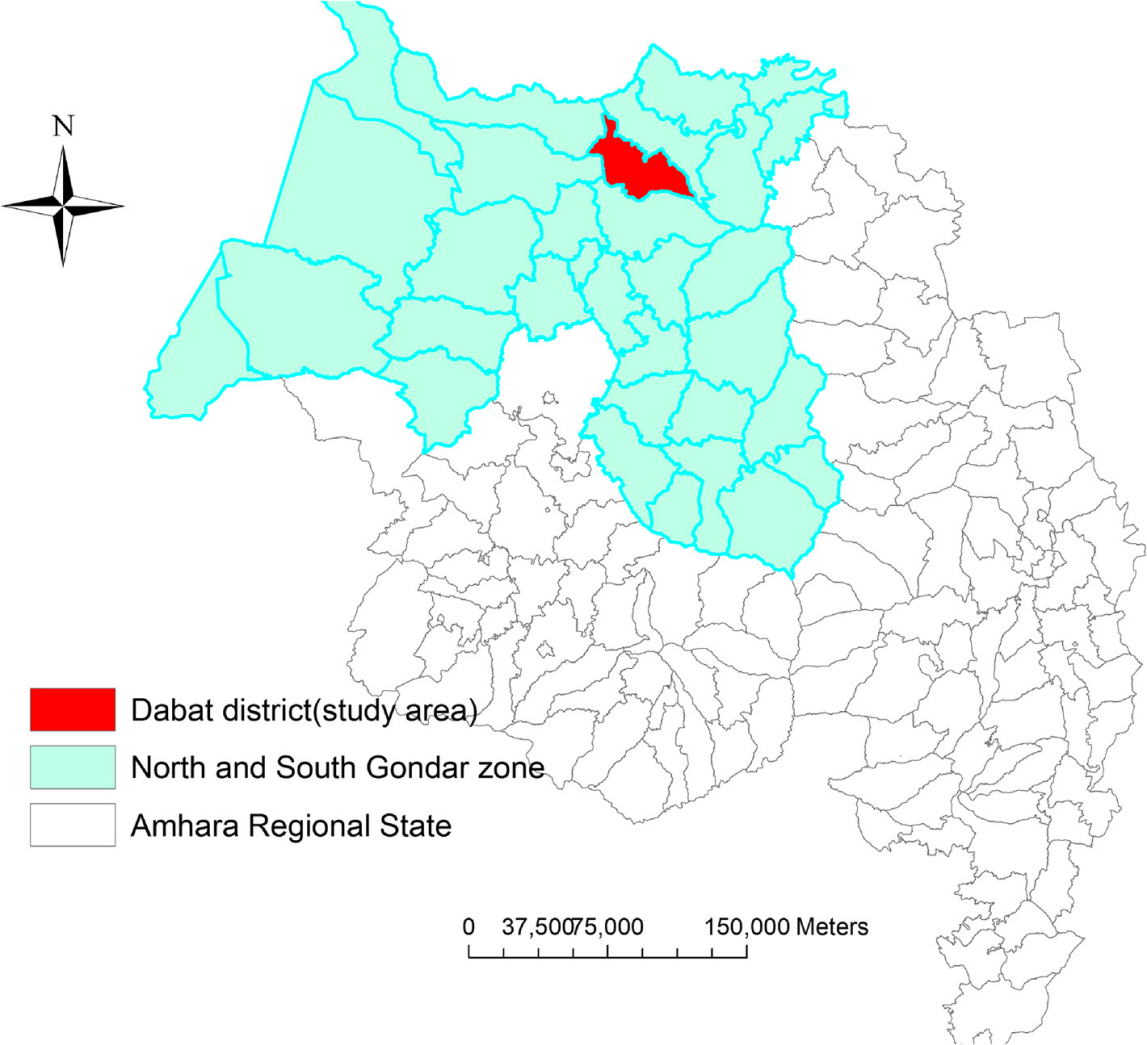
Map of the study area, northwest Ethiopia.

The study was conducted in Dabat district, which is a predominantly rural area with a population of 159,091. The area of the district is 1,187.93 sq km, and 133.9 population density [23] (Figure 1). The peak malaria transmission season in the study area is from September to December. Most malaria cases are due to *Plasmodium falciparum* infection.

### Sample size determination

Stat Calc module of Epi Info™ 7.1.3.3 software was used to compute the sample size with an assumption of 95% level of confidence, 80% power, odds ratio of 1.63, ratio of control to cases 2:1, and assuming that 6.9% of the controls travelled in the previous month [24]. A total of 1,934 (645 cases and 1,289 controls) was the planned sample size. During the actual data collection, a total of 1,644 patients (645 cases and 999 controls) were included in the study.

### Data

All patients who visited local health centres seeking treatment and who were permanent residents in the study district were included in the study. Patients who came from other districts were not included in the study. Daily malaria data were collected using passive surveillance system from patients who visited four local health facilities in the district from August 2012 to May 2013 (about 40 weeks). The data were collected by trained laboratory technicians working at each health facility laboratory unit, and were tested for malaria at the laboratory department.

A malaria case was defined as an individual from the study area who presented to the health facilities with malaria-like symptoms (fever, chills, severe malaise, headache; vomiting for children) and diagnosed either by microscopy, or rapid diagnostic test and microscopy together. Those patients who were negative for microscopy or rapid diagnostic test were regarded as patients without malaria.

Georeference coordinates were collected at village level using hand-held global positioning system (GPS). The *kebeles* (the lowest local administrative units) were also georeferenced with GPS. Each case and control was georeferenced to the location of the village in which the patient resided. A shape file with *kebele* and district boundaries and polygon shapes were obtained from the Central Statistical Agency of Ethiopia (CSA) [23] and each *kebele* was georeferenced to its geographic centroid. The patients’ addresses and data were crosslinked with *kebeles* and villages using the Arc GIS 10 software [25]. After completing the address and data crosslinked to villages and *kebeles*, each patient village and *kebele* was characterized by unique latitude and longitude location coordinates.

Individual information was collected using a pretested and structured questionnaire. The questionnaire comprised socio-economic characteristics, malaria preventive measures (insecticide-treated nets (ITNs) use, indoor residual spray (IRS)), and history of travel in the month preceding the interview, travel place, and the homestead surroundings.

### Cluster analysis

SaTScan™ software, version 9.1 [26] using the Kulldorf method was used to for cluster analysis. The discrete Bernoulli model [17,27], was used to analyse the spatial and temporal patterns of malaria transmission. The number of cases in each location had Bernoulli distribution and the model requires data with or without a disease. Patients with malaria were taken as cases and patients who were negative for malaria were taken as controls to fit the Bernoulli model for the purpose of this study. The binomial data were analysed with the purely temporal and spatial scan statistics.

Spatial scan statistics were used to explore the spatial pattern of malaria cases, used to test hypotheses relating to the processes that may have given rise to the observed distributions, and used to identify significant spatial clusters of malaria cases at village level. SaTScan software used a circular window moved systematically throughout the study area to identify significant clustering of malaria cases and centred on each of a number of possible locations throughout the study villages and for each location. Cluster analysis was performed with the default maximum spatial cluster size of <50% of the population and again with a smaller maximum cluster size of <25% to look for possible subclusters. Fifty per cent was specified as the upper limit, which allowed both small and large clusters to be detected and ignored clusters that contained more than 50% of the population. Likelihood ratio test was used to test the hypothesis that there were elevated malaria cases compared with the distribution outside the moving window. The window sizes and locations with the maximum likelihood were defined as the most likely cluster(s). Monte Carlo replications of the dataset determined the distribution and p-value of the most likely and secondary clusters. The aim was to detect villages with high numbers of malaria cases. A standard of ‘no geographical overlap’ was used to report secondary clusters. The P-value was created using a combination of standard Monte Carlo, sequential Monte Carlo and Gumbel approximation and used 999 replications of Monte Carlo [26].

### Statistical analysis

The malaria data were collected from patients seeking treatment at four local health facilities. These are typical Bernoulli data and modelled via logistic regression using Bayesian framework to identify the individual level factors. Let *Y*_*ij*_ and *p*_*ij*_ are the status and probability of *P. falciparum* or *Plasmodium vivax* infection, respectively, of all age patients *j* in village *i*. Assuming *Y*_*ij*_ has a Bernoulli distribution, *Y*_*ij*_ < *Bernoulli*(*p*_*ij*_) and modelled covariates *X*_*ij*_ and village-specific random effect Φ_i_ on the logit(p_ij_), that is logit(p_ij_) = X^T^_ij_β + Φ_i_, where β is the vector of regression coefficients [28].

Markov chain Monte Carlo simulation was used to estimate the model parameters using Gibbs sampling [29]. Two chain samplers with a burn in of 5,000 iterations were used. The deviance information criterion (DIC) was used to assess the goodness-of-fit of the full and reduced models [30] where the smaller DIC value was considered as the better model fit. The analysis was done using the Bayesian statistical software WinBUGS version 1.4.1 (MRC Biostatistics Unit, Cambridge, UK).

### Ethical issues

The protocol was approved by the University of Gondar Ethical Review Committees (IRB). Verbal and written consent was obtained from all patients. In the case of illiterate patients, thumb-printed consent was obtained after consent was read by trusted literates. Study procedures and purposes were explained to parents/guardians; their understanding was confirmed through an interview before written or thumb-printed consent was obtained. Interviews were completely voluntary and all data were kept confidential.

## Results

### Spatial distribution of malaria

A total of 1,644 patients presented with and without malaria. Of these, 645 cases of malaria were captured at the health facilities during the study period. Some 405 (62.8%) patients were diagnosed using microscopy and 240 (37.2%) of these diagnosed using rapid diagnostic test. *Plasmodium falciparum*, 355 (55.0%), was the higher species found, followed by *P. vivax*, 173 (26.8%), and all types, 117 (18.2%). Out of 645 malaria cases, 488 (75.7%) were from villages above 2,000 m above sea level, 66 (10.2%) were from villages between 1,750 and 2,000 m above sea level and 91 (14.1%) were from villages between 1,500 and 1,750 m above sea level.

**Figure 2:**
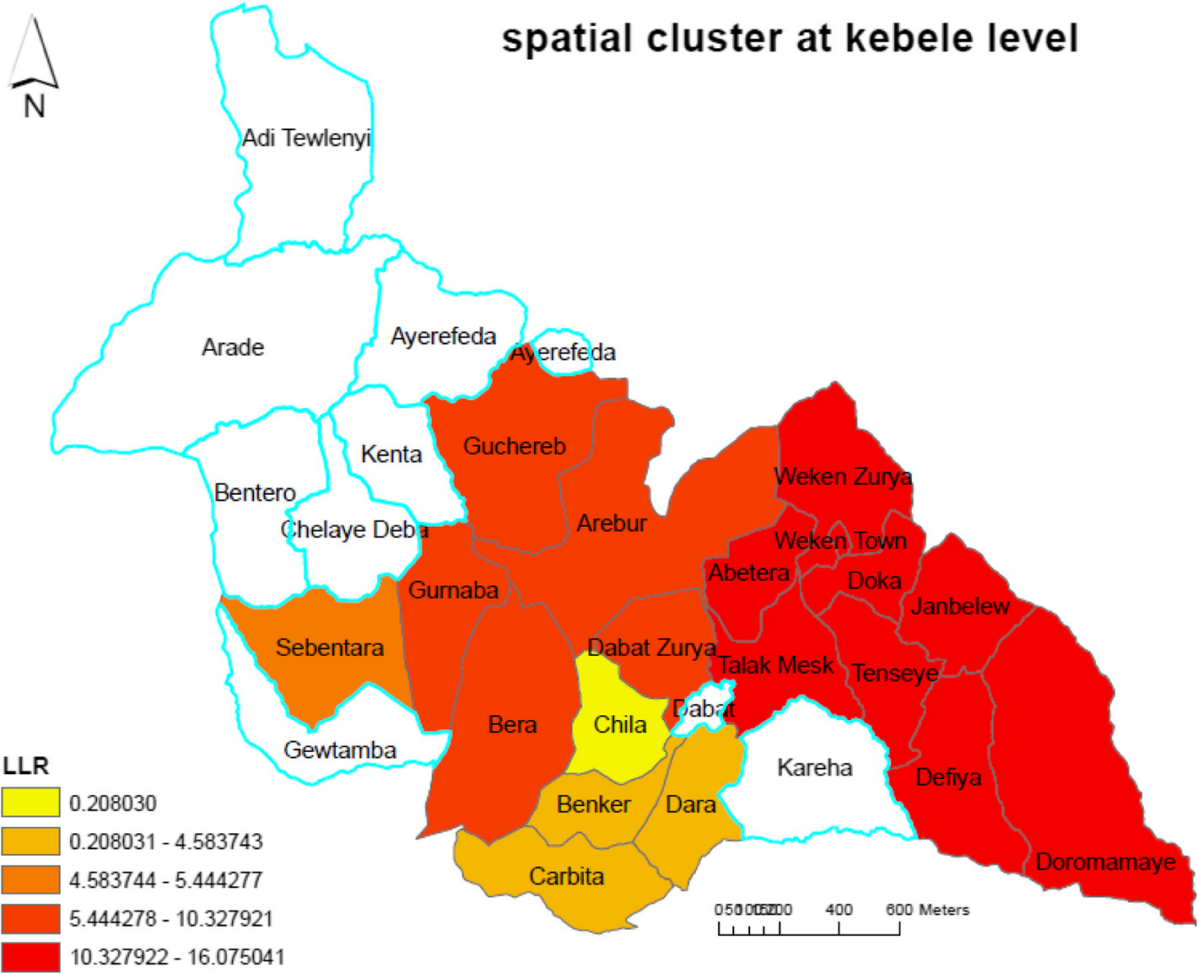
**Spatial clusters of malaria at *kebele *** **level based on log likelihood ratio (LLR), at Dabat district, northwest Ethiopia.**

**Figure 3:**
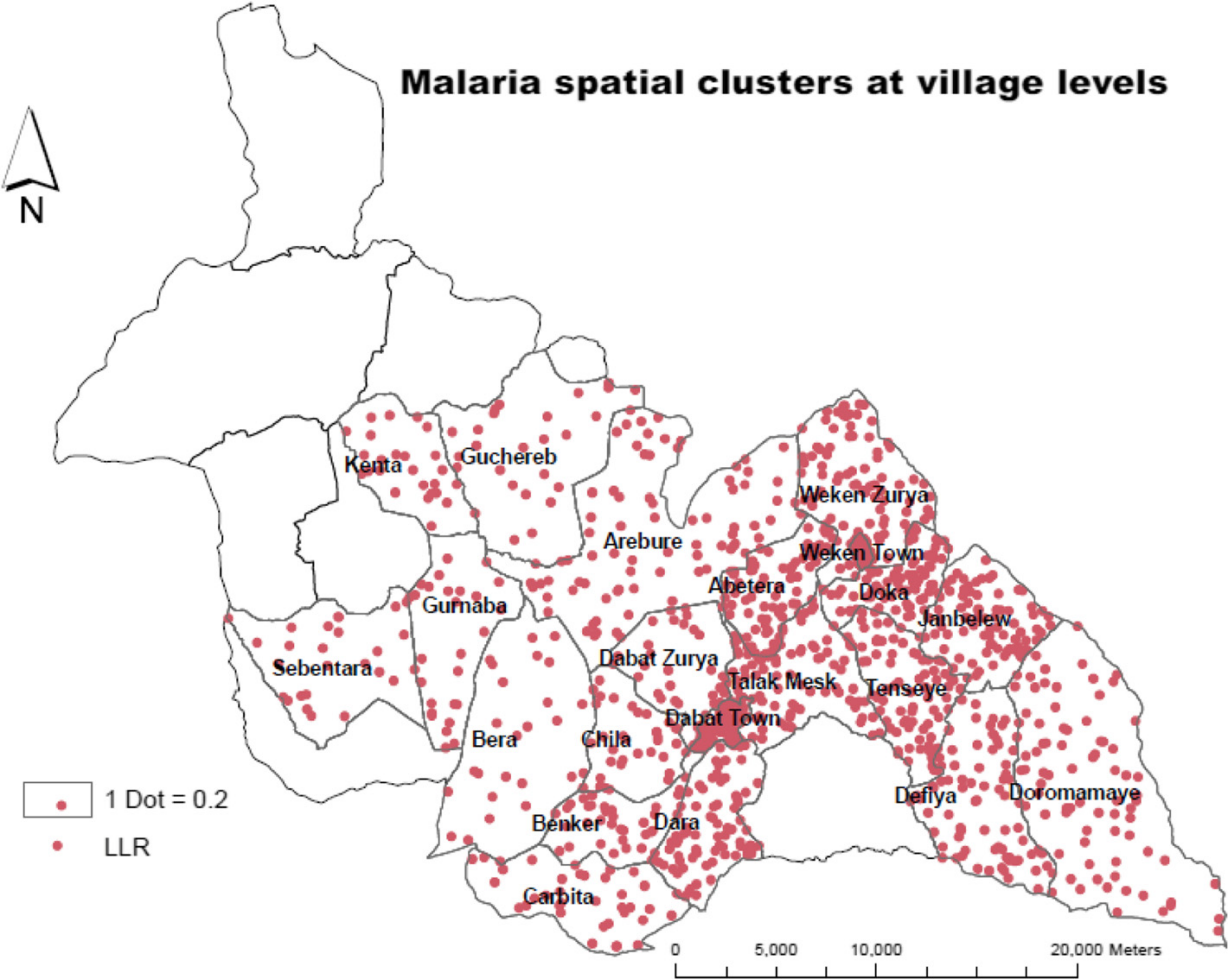
Spatial patterns of malaria transmission risks in high-altitude villages at Dabat district, northwest Ethiopia, 2013.

The most likely spatial clusters were detected in high-altitude *kebeles:* Janbelew, Tenseye, Doka, Defiya, Doromamaye, Wekentown, Talakmesk, Abetera, and Weken Zurya (LLR = 16.23, p < 0.001). These clusters accounted for 263 (40.8%) of malaria cases (Figure 2). The secondary clusters were found at *kebeles*: Carbita, Dara, and Benker (LLR = 4.58, p < 0.04) and accounted for 147 (22.8%) of malaria cases (Figure 3). Patients who travelled away from home to malaria-endemic areas were high in *kebeles* where malaria cases were found clustered.

**Figure 4:**
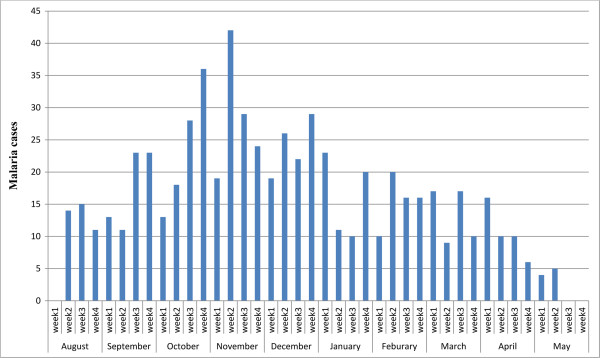
The weekly temporal pattern of malaria in high-altitude villages, 1 August, 2012 to 5 May, 2013.

Spatial scan statistics identified a total of 56 (the most likely and secondary) spatial clusters of malaria at village level. Of these, 39 villages were the most likely clusters (LLR = 15.62, p < 0.00001) and 17 villages were secondary clusters (LLR = 7.05, p < 0.03). The most likely spatial clusters of malaria identified in villages were from the same *kebeles* where malaria cases were spatially clustered (Figure 4). It means that the most likely clustered villages were within the most likely spatial clustered *kebeles*. Villages identified within spatial most likely clusters were located at altitudes above 2,000 m compared with villages outside the cluster and accounted for 302 (46.8%) of the malaria cases. The secondary clusters accounted for 113 (17.5%) of the total malaria cases and clustered in the villages located between 1,500 and 2,000 m highland fringes, which are prone to malaria epidemics (Figure 3).

### Temporal distribution of malaria

Of the total number of malaria cases, 384 (59.5%) were observed between September and December 2012. The peak months were November 110 (17.1%), October 107 (16.9%), and December 97 (15.0%). The majority, 354 (54.9%), of malaria infection was due to *P. falciparum* infections. September, October, November was the months of high malaria transmission. The peak weeks of malaria transmission were in November (week 2 and 4), and in October (week 3 and 4), and in December (week 2 and 4) (Figure 1).

Temporal scan statistics identified temporal clusters in all villages. The significant temporal clusters of malaria cases were from 1 August 2012 to 31 December 2012 (LLR = 17.874624, p < 0.001), followed by from 1 January 2013 to 28 February 2013 (LLR = 4.282231, p < 0.01). The secondary temporal clusters were found from 1 January 2013 to 28 February 2013 (LLR = 4.282231, p < 0.018).

### Factors associated with malaria clusters

Based on the DIC value, the full model was considered the best model. Travel away home, 95% credible interval (0.737; 4.154), males, 95% credible interval (0.8416; 4.829) and age above 15 years, 95% credible interval (0.061; 0.123) had statistically significant effect on malaria clusters at high-altitude villages (zero is not included in the 95% credible interval) (Table 1).

**Table 1:** Bayesian estimates for the model parameters for assessing the effect of factors on malaria clusters at village level, Dabat district, northwest Ethiopia, 2013

**Model**	**Parameters**	**Mean**	**Standard deviation**	**Median**	**95% credible interval**	**DIC**
**Model 1**						
Travel	β1	1.551	0.8075	1.274	0.985; 4.815	2097.520
**Model 2**						
Age	β 2	1.162	0.2452	1.128	0.6877; 1.613	2153.640
**Model 3**						
Sex	β3	1.367	0.1364	1.366	1.104; 1.638	2094.690
**Model 4**						
Travel	β1	1.299	0.4515	0.8825	0.03029; 1.136	
Age	β2	0.8393	0.2873	0.4212	0.01927; 0.7855	2197.930
**Model 5**						
Travel	β1	0.943	0.1114	0.9446	0.7293; 1.158	2026.020
Sex	β3	1.101	0.1414	1.103	0.8218; 1.388	
**Model 6**						
Age	β2	0.8033	0.1614	0.801	0.51; 1.118	2063.470
Sex	β3	1.294	0.1488	1.294	1.007; 1.589	
**Model 7**						
Travel	β1	1.22	0.7733	0.9866	0.737; 4.154	2018.270
Age	β2	0.021	0.0464	0.0183	0.061; 0.123	
Sex	β3	1.415	0.8817	1.159	0.8416; 4.829	

## Discussion

In this study, malaria transmission showed spatiotemporal clusters in high-altitude villages. Travel away home, males and age above 15 years had statistically significant effect on malaria spatiotemporal clusters in high-altitude villages.

The spatial cluster analysis identified high-risk villages in high altitudes. The most likely spatial clusters of malaria cases were detected in villages and *kebeles* located above 2,000 m above sea level. This may be due to health facilities being located in high altitudes and which are easily reached by patients who lived in high-altitude villages and patients who lived closer to low and mid-highlands may not easily get to these health facilities to seek malaria treatment. Further, the patients who travelled away from home to low-lying areas where malaria is endemic might be most probable to bring malaria infection to high-altitude villages. Peripheries of health facilities closer to low and mid-highlands had not malaria diagnosis and laboratory personnel. The secondary spatial clusters were found in villages and *kebeles* located between 1,500 and 2,000 m. Studies reported that the occurrence of malaria epidemics and transmission above 2,000 m [10,31], between 1,500 and 2,000 m [32,33] above sea level. The increased number of malaria cases in these altitudes may be attributed to the presence of elevated transmission effective vectors, or to the environment favouring breeding for the vectors. Other reasons may be due to the hilly geography of the villages where the people live on top of hills and move to lower altitudes for their agricultural activities.

The study indicated that the space distribution of malaria is closely related to the topography of the environment [8]. Unusual weather conditions of rainfall and temperatures [27,34] may favour malaria transmission in the area. Malaria prevention and control strategies have not yet prioritized in these areas [35]. In these areas, the people immunity is naïve and they are vulnerable to malaria epidemics. It is believed that the risk of malaria in the study villages may be closely related to the behaviour of villagers working and resting without protection, and mobility to lowland areas where malaria transmission is high. This shows that it is important to understand the spatial distribution of malaria at a finer geographic scale in high-altitude villages.

The majority of malaria cases occurred during September and December with peaks during November and accounted for the high proportion of total cases. The finding was consistent with another study [1]. The temporal variations may be related to variations in meteorological parameters [27] and could be utilized to effectively predict the incidence of malaria in high-altitude villages.

On the basis of this study, malaria transmission risks are in high-altitude villages located above 2,000 m. A patient who travels away from home to low-lying areas where malaria is endemic might have the most probable effect on malaria spatiotemporal patterns in high-altitude villages. Travel often increases exposure to infectious disease and can affect disease prevention and control efforts [36]. Travel has also contributed to the global spread of malaria during the history of humankind [37]. Non-immune individuals travelling from high, malaria-free altitudes to low-lying, malaria-endemic areas are predominantly susceptible to malaria infections if exposed to an infected mosquito, particularly in areas with high levels of transmission [4], and need to be educated about avoiding mosquito and other insect bites by using adequate preventive measures (ITNs and insect repellents).

The study focused on patients presenting at local health facilities during ten months, from August 2012 to May 2013, using a passive case detection method. This may be limited in showing the true picture of malaria at household and village level. The study was not sensitive to the locations of health facilities outside the study area; patients in certain *kebeles* and villages who sought care for malaria at facilities in adjacent districts would not be reflected in the dataset. Nevertheless, the passive case detection hinted at active case detection and identification of risk factors that cause spatiotemporal patterns of malaria risks in high-altitude villages. In addition, there is a loss of resolution by aggregating individual level data (obtained from health facilities) to the village level.

SaTScan and GIS-based spatial statistical techniques could provide an opportunity to clarify and identify malaria transmission risks within high-altitude villages, and help future investigations into environmental, climate and other factors responsible for increased malaria risks in high-altitude villages. The geographic visualization of malaria case distribution using these tools could help to understand malaria risk at village and *kebele* level and target interventions in high-risk areas.

In this study, only the associations between human population density/distribution and malaria cases in space-time and altitude were studied. Further studies to investigate the underlying causes of increased risk in the identified areas are recommended, including vector densities and distribution, vector infectivity, environmental and climatic factors, to have a more inclusive view of the malaria risk.

## Conclusion

The study identified spatial and temporal clusters of malaria in high altitudes villages located above 2,000 m. A patient who travels away from home to malaria-endemic areas might have the most effect on malaria clusters in high-altitude villages. These results provide further insights into identifying the true picture of malaria clusters and causes at villages and enable the efficient and timely spatial targeting of preventive and control measures against the disease in high altitudes villages where people have low immunity.

## Competing interests

The authors declare that they have no competing interests.

## Authors’ contributions

Conception and design of the work, acquisition of data, analysis and interpretation of data was done by KA, AW and YB. Drafting the article, revising it critically for intellectual content, and final approval of the version to be published was done by KA, AW and YB. All authors read and approved the final manuscript.
